# Editorial: Microbial Interactions With Nanomaterials in the Environment and Their Application

**DOI:** 10.3389/fmicb.2022.850141

**Published:** 2022-03-24

**Authors:** Fayuan Wang, Linghao Zhong, Yucheng Wu, Shiying He

**Affiliations:** ^1^College of Environment and Safety Engineering, Qingdao University of Science and Technology, Qingdao, China; ^2^Pennsylvania State University at Mont Alto, Mont Alto, PA, United States; ^3^Institute of Soil Science, Chinese Academy of Sciences, Beijing, China; ^4^Institute of Agricultural Resources and Environment, Jiangsu Academy of Agricultural Sciences, Nanjing, China

**Keywords:** nanomaterials, microbial activity, microbial community, ecotoxocity, pollution treatment

Thanks to recent advances in multiple fronts such as material science and nanotechnology, the applications of nanoparticles (<100 nm) are now expanded into numerous fields. The increased applications, undoubtedly and unfortunately, are accompanied by the detection of elevated levels of nanoparticles in the environment (Zhang et al., 2016; Zhong et al., 2020). Concerns have been raised about the fate and behavior of nanoparticles, and yet, no consensus has been reached on their biological impacts on the organisms in the environment. Microorganisms are active components in the environment and are sensitive to micro-niche variations. What becomes more intriguing is that nanoparticles can have both positive and negative impacts on the environment (Xiao et al., 2018; Zhang et al., 2020). To settle the arguments, an understanding of the mechanism of nanoparticle-environment interaction will surely be very helpful. This knowledge can also guide designs to switch purposefully enable or disable certain interactions. To stimulate and congregate discussions on this topic, we present the Research Topic “Microbial Interactions with Nanomaterials in the Environment and their Application” as a platform for researchers to join forces in furthering our understandings of how environmental microorganisms respond to different nanomaterials.

In this Research Topic, two articles focused on the effects of nanoparticles on soil ecosystems. Liu et al. investigated the transformation of selenium nanoparticles (SeNPs) and selenite in soil. Comparing the different impacts they brought to soil microorganisms, the authors demonstrated that while SeNPs showed longevity and stability in soil, selenite transformed into various more chemically stable states. Because of their slow-release effect, SeNPs benefit soil microorganisms for a longer period of time with lower toxicity, and consequently, less disturbance. Overall, this study illustrated how SeNPs can have an edge on replacing traditional Se fertilizer for Se-enriched agricultural products. Contrary to the positive impacts from SeNPs, a mixed impact was reported in the second article. Zhang et al. studied how Ag nanoparticles (AgNPs) affect denitrification potential and microbial communities in paddy soil. They found that low doses of AgNPs had no significant effects on the denitrification rate. The rate was significantly stimulated only at high doses. Authors attributed these findings to the tolerance of soil nitrate reductase gene even at high AgNPs doses, and they identified relevant microbial phylotypes responsible for the stimulation. Hence, it is understandable when authors expressed their cautious optimism about AgNPs and called for careful risk assessment when applying AgNPs in agroecosystems. In the third report, Liu et al. turned their attention to honeybees. Honeybees provide essential pollination services for agricultural ecosystems and valuable apiary products for human nutritional needs, but they are highly susceptible to nano-La_2_O_3_, which is commonly used in agricultural practices. The authors assessed the effects of nano-La_2_O_3_ on the health of honeybees. The results showed nano-La_2_O_3_ led to dysbiosis of honeybee gut bacterial communities and subsequently exerted a dose-depend detrimental effect on honeybee physiology. Authors also observed enrichment of pathogenic *Serratia* and *Frischella* alongside the exposure of honeybees exposure to nano-La_2_O_3_. In their conclusion, the authors suggested the adverse impact of nano-La_2_O_3_ on the health of honeybees was caused by pathogen enrichment and gut dysbiosis.

The final two articles investigated the applications of nanoparticles in ag practices. In their article, Sarangapani et al. demonstrated the potency of thymol-loaded chitosan nanoparticles (TCNPs) to inhibit bacterium Xanthomonas campestris pv. campestris (Xcc) growth by “disruption of the membrane integrity and reduction of the cell viability.” This study adds an attestation to the consensus of research findings in this area. However, it is interesting that the authors note that upon TCNPs treatments Xcc produces a significant shift in VOCs profile, indicating possible different metabolic pathways. As the authors rightfully pointed out, a more in-depth analysis of the shift is needed as such knowledge can shed light on the mechanism of the function of TCNPs. We are looking forward to more such investigations in the future. In the last report in this Research Topic, Bastian et al. introduced a non-destructive approach to specifically capture eukaryotic cells. The procedure involves first grafting of superparamagnetic nanoparticles onto targeted micro-eukaryotic cells using yeast (*Saccharomyces cerevisiae*) as a model, followed by isolating the eukaryotic cells from an artificial mixture of bacterial cells by using a micro-magnet array. Such a method combines hybridization chain reaction (HCR) and magnetic *in situ* hybridization (MISH), proven to be a practical and promising technology for the capture of specific microeukaryotes. Allowing an efficient separation of specific cells in more complex cellular mixtures, this method is bound to open up innumerable opportunities in ecological science investigations.

Through these studies, it becomes obvious that a generalized conclusion of nanoparticle-environment interactions is not only impossible but also unrealistic. Due to the delicate environmental conditions at all levels varying from molecular biology level to ecology level, the question to be asked should not be if the nanoparticle is beneficial or detrimental to an environment, as there is no guarantee that a conclusion reached in one environment and condition can be replicated at other locations. This Research Topic, therefore, highlights a notable challenge to the scientific community to continue elucidating the underlying mechanisms of nanoparticle-environment interactions, so new guidelines and tools can be developed to steward a sustainable ecosystem. However, with already published knowledge of different nanoparticles as the single factor, one particular area that can be imminently beneficial, both scientifically and practically, is the investigation of composite (nanoparticle mixtures or nanoparticle-chemical mixtures) applications.

## Author Contributions

All authors drafted, corrected, and approved the editorial for publication.

## Conflict of Interest

The authors declare that the research was conducted in the absence of any commercial or financial relationships that could be construed as a potential conflict of interest.

## Publisher's Note

All claims expressed in this article are solely those of the authors and do not necessarily represent those of their affiliated organizations, or those of the publisher, the editors and the reviewers. Any product that may be evaluated in this article, or claim that may be made by its manufacturer, is not guaranteed or endorsed by the publisher.
